# Proteomic profiles in inclusion body myositis and polymyositis with mitochondrial pathology

**DOI:** 10.1186/s40478-026-02243-9

**Published:** 2026-02-04

**Authors:** Felix Kleefeld, Christina B. Schroeter, Donya Abdennebi, Vera Dobelmann, Sara Walli, Andreas Roos, Ute Distler, Stefan Tenzer, Tobias Bopp, Paula Quint, Linda-Isabell Schmitt, Markus Leo, Tim Hagenacker, Iago Pinal-Fernandez, Maria Casal-Dominguez, Andrew L. Mammen, Corinna Preuße, Lorenzo Maggi, Alexander Mensch, Sven G. Meuth, Werner Stenzel, Tobias Ruck, Christopher Nelke

**Affiliations:** 1 Department of Neurology, BG University Hospital Bergmannsheil, Ruhr University Bochum, Bochum, Germany; 2 Heimer Institute for Muscle Research, BG University Hospital Bergmannsheil, Bochum, Germany; 3 Charité-Universitätsmedizin Berlin, Corporate Member of Freie Universität Berlin, Berlin Institute of Health (BIH), Department of Neurology, Humboldt Universität Zu Berlin, Charitéplatz 1, 10117 Berlin, Germany; 4 Department of Neurology, Medical Faculty and University Hospital Düsseldorf, Heinrich Heine University, Düsseldorf, Germany; 5 Institute of Immunology, University Medical Centre of the Johannes-Gutenberg University, Mainz, Germany; 6 Department of Neurology, Center for Translational Neuro- and Behavioral Sciences (C-TNBS), University Medicine Essen, Hufelandstr. 55, 45147 Essen, Germany; 7 Muscle Disease Unit, National Institute of Arthritis and Musculoskeletal and Skin Diseases, National Institutes of Health, Bethesda, MD 20892 USA; 8 Department of Neurology, Johns Hopkins University School of Medicine, Baltimore, MD 21205 USA; 9 Charité-Universitätsmedizin Berlin, Corporate Member of Freie Universität Berlin, Berlin Institute of Health (BIH), Department of Neuropathology, Humboldt-Universität Zu Berlin, Charitéplatz 1, 10117 Berlin, Germany; 10 Foundation IRCCS, Neurological Institute Carlo Besta, Milan, Italy; 11 Department of Neurology, University Medicine Halle, 06120 Halle (Saale), Germany

## Abstract

**Background:**

Idiopathic inflammatory myopathies (IIMs) are autoimmune muscle diseases with distinct clinical, histopathological, and molecular features. Among them, inclusion body myositis (IBM) is refractory to immunotherapy and characterized by combined inflammatory and degenerative changes. Polymyositis with mitochondrial pathology (PM-Mito) has been proposed as a prodromal stage of IBM, but molecular profile underlying this spectrum remains poorly defined.

**Methods:**

Skeletal muscle biopsies from 38 IBM, 14 PM-Mito, 5 anti-synthetase syndrome (ASyS), 3 dermatomyositis (DM), 5 immune-mediated necrotizing myopathy (IMNM), and 7 non-diseased controls (NDC) were analyzed by label-free mass spectrometry and validated by bulk RNA sequencing. Dimensionality reduction was performed using sparse Partial Least Squares Discriminant Analysis (sPLS-DA), followed by differential protein analysis.

**Results:**

IBM exhibited a homogeneous and distinct proteomic signature compared with other IIM subtypes, driven by upregulation of MHC class I (e.g. HLA-A) and II (e.g. HLA-DRB1, CD74) molecules, and cytoskeletal proteins (e.g. PDCL3). Comparing IBM to other types of IIM, we also detected increased level of specific histone variants (e.g. HIST2H2AA3, H1FX). Enrichment analysis of the differential proteins underscored increased antigen presentation and T-cell–mediated immunity pathways, with concomitant depletion of mitochondrial respiratory chain, RNA processing, and oxidative phosphorylation components in IBM. PM-Mito shared a proteomic profile with IBM with reduced MT-ND2 levels and increases in lipid storage regulator PLIN1 and extracellular matrix protein COL14A1, among others. In contrast to IBM, PM-Mito preserved type 2 myofiber markers (e.g. MYH2). A specific protein change to PM-Mito was an increase in the cytochrome c oxidase subunit III (MT-CO3), implicating mitochondrial remodelling. Transcriptomic analysis validated the proteomic changes in *COL14A1*, *IGLL4*, *PLIN1*, *MT-ND2*, *SMDT1*, and *TIMM21*, all of which were shared between IBM and PM-Mito.

**Conclusion:**

IBM exhibits a unique proteomic landscape distinct from other IIMs. The overlap with PM-Mito suggests that these conditions share molecular features, supporting an interpretation that places PM-Mito in the broader spectrum of IBM. Novel protein markers, including histone variants and cytoskeletal regulators, highlight potential pathways for future research. These findings underscore the need for longitudinal studies exploring therapeutic targets in early disease stages.

**Supplementary Information:**

The online version contains supplementary material available at 10.1186/s40478-026-02243-9.

## Introduction

Idiopathic inflammatory myopathies (IIMs) are a heterogeneous group of autoimmune disorders affecting skeletal muscle, with some subtypes also causing extramuscular complications. These IIM subtypes include dermatomyositis (DM), anti-synthetase syndrome (ASyS), immune-mediated necrotizing myopathy (IMNM), and inclusion body myositis (IBM) [1], with polymyositis with mitochondrial pathology (PM-Mito) proposed as an additional variant [2–5]. Although DM, ASyS, and IMNM share overlapping features such as proximal muscle weakness, IBM is distinct in its insidious onset, poor immunotherapy response, and a combination of inflammatory and degenerative histopathological markers (e.g., rimmed vacuoles, protein aggregates, and CD8⁺ T cell–predominant endomysial inflammation) [6].

Mitochondrial dysfunction plays a central role in IBM contributing to both muscle weakness and chronic inflammation [3, 7]. Recent data suggest substantial histopathological and molecular similarities between IBM and PM-Mito, implicating that PM-Mito may represent an early stage of IBM. Indeed, while data supports the inclusion of PM-Mito in the IBM spectrum [6], there are clinical and histopathological differences between these entities. As such, patients with PM-Mito may demonstrate a transient clinical response to immunosuppression [5] and atypical clinical and histopathological characteristics. Concurrently, patients with PM-Mito progress to clinically or histopathologically confirmed IBM in 50% to 90% of cases, depending on the study cohort [3–5]. Together, these data suggest that PM-Mito may represent a precursor state to IBM, although further research is needed to better understand and characterize the disease spectrum.

In this study, we conducted a comprehensive proteomic analysis to characterize the molecular landscape of IBM, comparing it with DM, ASyS, and IMNM. Additionally, we included PM-Mito as a potential precursor to IBM and studied how this disease spectrum progresses. Although previous proteomic studies on muscle biopsies [8], serum samples [9], or isolated organelles [10] have yielded valuable insights, they were often limited to single IIM entities or small cohorts. These studies have underlined that IBM is characterized by high levels of antigen-presenting proteins, e.g., components of the major histocompatibility (MHC) complexes I and II, aligning with histopathological findings [8]. PM-Mito, to our knowledge, has not been studied on the proteomic level.

Here, we describe distinct molecular changes to the proteome in IBM and PM-Mito. Both diseases exhibit distinct molecular profiles but also highlight a shared mitochondrial and immune phenotype that differentiates these conditions from other IIM subtypes.

## Methods

### Sample collection and clinical characterization

Skeletal muscle biopsies were collected from patients diagnosed with clinico-pathologically defined IBM according to the 2011 ENMC criteria [11] (n = 38), PM-Mito (n = 14), ASyS (n = 5), DM (n = 3), and IMNM (n = 5) for analysis through label-free mass spectrometry-based proteomics. Biopsies were obtained from the quadriceps muscle for diagnostic purposes in all cases and cryopreserved at − 80 °C. All samples were obtained with patient consent and stored in the biobank at the Department of Neurology, University Hospital Düsseldorf, and at the Department of Neuropathology in Berlin, in compliance with local Ethics Committee guidelines (Berlin: EA2/107/14; Düsseldorf: 2016-053-f-S and 2021-1417). The analysis of the PM-Mito cohort is a re-analysis of our previously published cohort from 14 PM-Mito to 10 IBM patients and seven non-diseased controls (NDC), as described in [12].

Detailed clinical data and medical histories were collected for all patients at the time of study inclusion. This encompassed a comprehensive neurologic examination, as well as a general physical examination, which included an assessment for skin abnormalities.

### Bulk RNA sequencing

Bulk RNA sequencing (RNA-seq) was conducted on frozen muscle biopsy samples of IBM and PM-Mito patients as previously described [12–15]. For this study, we have re-analysed our previously generated data. The RNA isolation, and the bulk RNA-sequencing were described in our previous publication. Control samples consisted of histologically normal muscle biopsies obtained from three sources: the Johns Hopkins Neuromuscular Pathology Laboratory (n = 12), the University of Kentucky Skeletal Muscle Biobank (n = 8), and the National Institutes of Health (n = 13). The Johns Hopkins samples were acquired for clinical purposes and showed no histopathological abnormalities; the remaining control samples were collected from healthy volunteers. For this study, we re-analysed our data, focusing on specific genes of interest as validation for our proteomics data. The pre-processed data, as previously reported, was used for this approach. We queried specific genes of interest in the dataset and reported their distribution across groups. Groups were compared by ANOVA without post-hoc correction of statistical significance. The raw data is available upon reasonable request from the corresponding authors.

### Protein extraction, mass spectrometry workflow, and proteomic analysis

Proteomic analysis was conducted using label-free mass spectrometry-based proteomics. The analysis was performed at the Core Facility for Mass Spectrometry & Proteomics of the University Medical Centre of the Johannes-Gutenberg University Mainz, Germany.

For protein extraction, 200 µL of an urea-based lysis buffer (7 M urea, 2 M thiourea, 5 mM dithiothreitol (DTT), 2% (w/v) CHAPS) was added to the muscle biopsies. Lysis was promoted by sonication at 4 °C for 15 min using a Bioruptor (Diagenode, Liège, Belgium). Afterwards, the protein concentration was determined using the Pierce 660 nm protein assay (Thermo Fisher Scientific) according to the manufacturer´s protocol. Proteins (corresponding to 20 µg) were digested using a modified filter-aided sample preparation (FASP) protocol as detailed before [16, 17]. In brief, samples were transferred onto spin filter columns (Nanosep centrifugal devices with Omega membrane, 30 kDa MWCO; Pall, Port Washington, NY). Afterwards, detergents were removed washing the samples (membrane) three times with a buffer containing 8 M urea. After reduction and alkylation by DTT and iodoacetamide (IAA), excess IAA was quenched with DTT and the membrane washed three times with 50 mM NH_4_HCO_3_. Afterwards, proteins were digested overnight at 37 °C with trypsin (Trypsin Gold, Promega, Madison, WI) using an enzyme-to-protein ratio of 1:50 (w/w). After digestion, peptides were recovered by centrifugation and two additional washes with 50 mM NH_4_HCO_3_. Combined flow-throughs were acidified with trifluoroacetic acid (TFA) to a final concentration of 1% (v/v) TFA and lyophilized. Purified peptides were reconstituted in 0.1% (v/v) formic acid (FA) for LC–MS analysis.

LC–MS analyses were performed on an Evosep One system (Evosep, Odense, Denmark) coupled a timsTOF HT mass spectrometer (Bruker Corporation). Peptides (corresponding to 300 ng) were loaded onto Evotips (Evosep, Odense, Denmark) according to manufacturer´s instructions. Peptides were separated using the “60 samples per day” method resulting in a total analysis time of 24 min per sample. For peptide separation, an 8°cm reverse-phase C18 column was used (PepSep™, 8 cm × 150 µm 1.5 µm), which was heated to 40 °C.

Samples were analyzed in positive mode ESI (electrospray ionization)-MS applying parallel accumulation serial fragmentation enhanced data-independent acquisition (diaPASEF [18]). A Captive Spray source (Bruker Corporation) equipped with a 20 µm zero dead volume (ZDV) emitter was used for ionization applying a capillary voltage of 1500 V. The dual TIMS was operated at a fixed duty cycle close to 100% using equal accumulation and ramp times of 100 ms each, spanning a mobility range from 1/K_0_ = 0.6 to 1.6 Vs  cm^−2^. We defined 32 × 35 Th isolation windows from *m/z* 298 to 1387 resulting in 4 diaPASEF scans per TIMS cycle and an overall cycle time of 0.9 s. The collision energy was ramped linearly as a function of the mobility from 59 eV at 1/K_0_ = 1.3 Vs cm^−2^ to 20 eV at 1/K_0_ = 0.85 Vs cm^−2^. MS1 and fragment ion spectra were recorded with a mass range spanning from *m/z* 100–1700.

### Bioinformatics and statistical analysis

#### Data processing

MS raw data were processed using DIA-NN (version 1.8.1) [19] applying the default parameters for library-free database search. Data were searched using a custom compiled database containing the UniProtKB entries of the human reference proteome and a list of common contaminants (version release April 2022, 20,361 entries). For peptide identification and in-silico library generation, trypsin was set as protease allowing one missed cleavage. Carbamidomethylation was set as fixed modification and the maximum number of variable modifications was set to zero. The peptide length ranged between 7–30 amino acids. The precursor *m/z* range was set to 300–1800, and the product ion *m/z* range to 200–1800. As quantification strategy we applied the “Robust LC (high precision)” mode with RT-dependent median-based cross-run normalization enabled. We used the build-in algorithm of DIA-NN to automatically optimize MS2 and MS1 mass accuracies and scan window size. Peptide precursor FDRs were controlled below 1%. Proteins had to be identified by at least two peptides.

Proteomics data were pre-processed using a two-step imputation strategy tailored to missing value patterns. Proteins missing in more than 70% of samples were removed. Remaining proteins with low missingness (< 20%) were imputed using K-nearest neighbor (KNN) imputation, while proteins with moderate missingness (20–80%) were imputed using a Perseus-like approach, involving downshifted random sampling from a log-normal distribution. Zero-variance proteins were excluded.

**Fig. 1:**
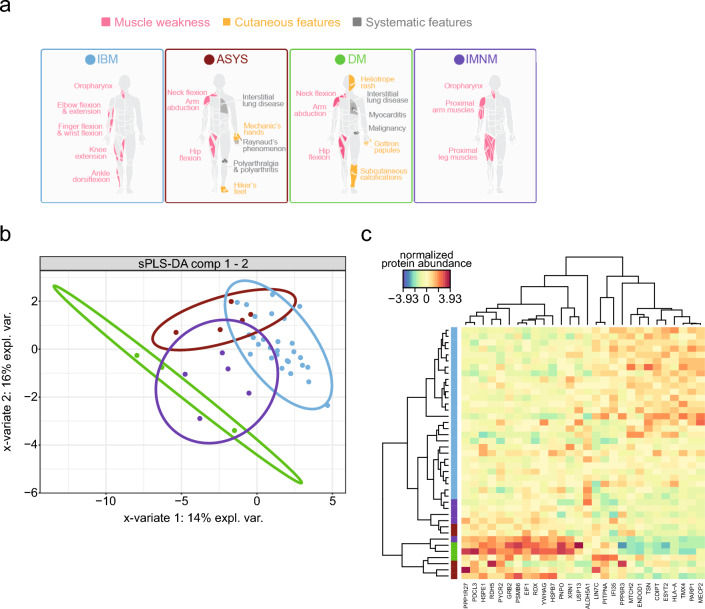
Sparse partial least-squares discriminant analysis (sPLS-DA). **a** Overview of IBM (blue), ASyS (red), DM (green), and IMNM (purple). The models illustrate the localization of muscle weakness (pink), cutaneous features (yellow), and systemic features (grey) for each disease. **b** Balanced error rates with each colour representing a different number of components (comp). The number of selected features kept in the analysis is represented on the x-axis, and the overall error on the y-axis. **d**,** e** Sample plots from sPLS-DA for the first and second component (**c**), the first and third component (**d**) and the second and third component (**e**). The dots represent the obtained samples with IBM (n = 18), ASyS (n = 5), DM (n = 3), and IMNM (n = 5). The percentage of explained variance (expl. var.) captured for the components is shown on the x- and y-axis. **f** Clustered Image Map (CIM) of the data selected by sPLS-DA. Patients are depicted in rows, proteins in columns. The red and blue colours indicate the scaled protein levels. The dendrograms indicate the clustering of proteins and proteins, respectively

For supervised multivariate analysis, sparse Partial Least Squares Discriminant Analysis (sPLS-DA) was performed using the mixOmics package (v6.31) [20]. The number of components and variables to retain (keepX) was tuned using fivefold cross-validation repeated 10 times. Sample discrimination and feature loadings were visualized via individual and loading plots, and heatmaps with group-specific side colors were generated. This approach extends classical PLS by incorporating Lasso penalization, effectively reducing the number of features (i.e., proteins) to those with the highest value for differentiating between groups. We configured the sPLS‑DA model to maximize the differences among IIM subtypes, tuning it based on the balanced error rate (BER) to determine the optimal number of discriminating proteins (Fig. 1b). Following feature selection, we performed dimensional reduction using the proteins identified by the sPLS‑DA model and evaluated the first three components. Components 1 and 2 accounted for a substantial portion of the variance (14% and 16%, respectively), while components 3 and 4 contributed to a lower degree (3.4% and 2.8%, respectively). The classification performance of the model was assessed by one-Vs-rest testing for each group. We report the Area Under the Receiver Operating Characteristic Curve (AUROC) for each group.

Differential protein analysis was conducted using the ANOVA test, followed by false discovery rate (FDR) correction via fdrtool with significance set to q ≤ 0.05. Proteins were ranked by q-value and log2 fold change. Results were visualized using volcano plots. For functional interpretation, Gene Set Enrichment Analysis (GSEA) was performed on ranked protein lists using the clusterProfiler package (v3.10), with annotations from the org.Hs.eg.db database (v3.22) and biological process (BP) and cellular components (CC) ontology [21].

#### Statistical analysis

Statistical analysis was performed using R (v4.5.1). Data were presented as median with IQR, mean ± standard deviation (SD), as absolute (n) or relative frequencies (%). Normality was evaluated using the Shapiro–Wilk test. Differences between the two groups were analysed using the Student’s t-test. ANOVA test was used for multiple groups. *p* > 0.05 was classified as not significant, *p* < 0.05 as significant (*), *p* < 0.01 (**), *p* < 0.001 (***).

## Results

### Proteomic characteristics of idiopathic inflammatory myopathy subtypes

The demographic and clinical characteristics of our patient cohort are summarized in Table 1. Clinically, patients with IBM, PM‑Mito, and IMNM demonstrated no extramuscular involvement. Specifically, IMNM patients predominantly exhibited proximal muscle weakness, whereas IBM patients showed a distinct pattern with pronounced weakness in the finger flexors, knee extensors, and quadriceps muscle (Fig. 1a). In contrast, PM‑Mito patients presented a more variable phenotype, with symptoms ranging from myalgia to either proximal, distal, or combined muscle weakness. Meanwhile, patients with ASyS and DM shared overlapping features, including muscle weakness, cutaneous manifestations, and systemic symptoms, as has been previously described [22].

**Table 1:** Demographic data of the IIM patient cohort

	NDC	IBM	PM-Mito	ASYS	IMNM	DM
Cross-sectional cohort						
Number	–	28	–	5	5	3
Age at biopsy (Median, range)	–	74 (54 to 87)	–	58 (28 to 65)	65 (41 to 81)	32 (21 to 48)
Gender (male/female)		16/12		3/2	3/2	1/2
Previous treatments (Number of patients, %)	–	14 (50.0%)	–	5 (100.0%)	5 (100.0%)	5 (100.0%)
IVIG	–	14 (50.0%)	–	3 (60.0%)	3 (60.0%)	0 (0%)
Cortisone	–	2 (7.1%)	–	4 (80.0%)	4 (80.0%)	3 (100.0%)
MTX	–	0 (0%)	–	1 (20.0%)	0 (0%)	1 (20.0%)
Azathioprine	–	0 (0%)	–	0 (0%)	0 (0%)	1 (20.0%)
CK in U/I (Median, range)	–	236 (84 to 492)	–	488 (155 to 921)	723 (231 to 1253)	337 (153 to 723)
Antibodies (Number of patients, %)	–	Anti-cN1A (46.4%)	–	Anti-Jo1 (60.0%), anti-PL7 (40.0%)	Anti-SRP (40.0%), anti-HMCR (40.0%)	Anti-Mi2 (100.0%)

Longitudinal cohort						
Number	7	10	14	–	–	–
Age at biopsy (Median, range)	61 (47 to 77)	68 (61 to 83)	63 (48 to 81)	–	–	–
Gender (male/female)			3/14			
Previous treatments (Number of patients, %)	0 (0.0%)	7 (70.0%)	11 (78.6%)	–	–	–
IVIG	0 (0.0%)	7 (70.0%)	3 (21.4%)	–	–	–
Cortisone	0 (0.0%)	1 (10.0%)	8 (57.1)	–	–	–
MTX	0 (0.0%)	0 (0.0%)	4 (28.5%)			
CK (Median, range)	68 (47 to 98)	486 (193 to 912)	514 (207 to 1706)	–	–	–
Antibodies	None	Anti-cN1A (30.0%)	Anti-cN1A (7.1%)	–	–	–

Abbreviations: CK = creatine kinase, IVIG = intravenous immunoglobulins, MTX = methotrexate

To understand the differences between these subtypes and reduce the complexity of the dataset, we utilized an sPLS‑DA model. For the final model, class-wise discrimination was quantified using one-Vs-rest cross-validated AUROC (mean ± SD across repeats) yielding the following values: ASYS 0.760 ± 0.086, DM 0.698 ± 0.064, IBM 0.785 ± 0.055, and INMN 0.699 ± 0.089. Classification performance was assessed by repeated M-fold cross-validation. Using the prediction distance, the overall misclassification error rate was 0.305 ± 0.042 and the balanced error rate (BER) was 0.610 ± 0.080 (mean ± SD across repeats). The final model included a total of 28 proteins. We examined the dimensional reductions of components 1 and 2, that explained a total of 30% of the variance in the dataset (Fig. 1b). Among others, IBM demonstrated an overlap with ASyS across components. Next, we examined the specific proteins that contributed most to the discrimination among the subtypes (Fig. 1f). IBM samples exhibited a highly homogeneous and distinct proteomic profile compared to the other IIM subtypes.

These findings indicate that the proteomic profiles of IIM subtypes are distinct, with IBM displaying a unique signature marked by specific downregulation and upregulation of proteins. The differences reinforce the distinct clinical and pathological characteristics observed in this subtype compared to other forms of IIM.

### Biological patterns in inclusion body myositis

**Fig. 2:**
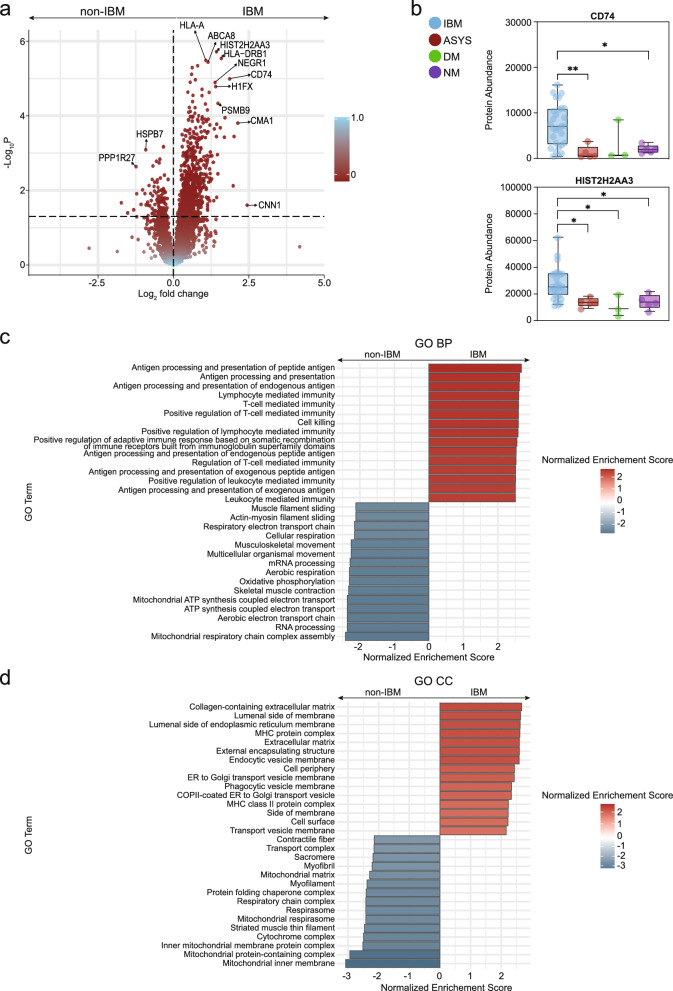
Differential proteins in IBM and controls **a** Volcano plot comparing IBM to non-IBM patients. ASyS, DM and IMNM patients were grouped as non-IBM. The y-axis represents the − log_10_ *p*-value and the x-axis the log_2_ fold change. Groups were compared by the Student’s T-test with Bonferroni correction for multiple testing. **b** Box-plots indicating the individual protein levels for each patient across IIM groups for exemplary proteins. The whiskers indicate the minimum and maximum values with all patients shown. **c** Gene Set Enrichment Analysis (GSEA) of the Biological Processes (BP) database of the differential proteins comparing IBM to non-IBM patients. All proteins with a p-value below 0.05 after multiple correction were considered. The Normalized Enrichment Score is shown on the x-axis. **d** GSEA for the Cellular Components (CC) database. Groups were compared by the ordinary one-way ANOVA test. *p* < 0.05 *, *p* < 0.01 **. *p* ≥ 0.05 not significant

To elucidate the overall proteomic differences between IBM and other IIM subtypes, we first grouped all non-IBM cases and identified differentially expressed proteins (DEPs) between these two cohorts (Fig. 2a). This analysis revealed a substantial number of DEPs that robustly distinguished IBM from non-IBM samples. Notably, proteins belonging to the major histocompatibility complexes (MHC) were among the most highly upregulated in IBM. For instance, HLA-A (belonging to MHC class I) and HLA-DRB1 (belonging to MHC class II) were markedly increased, mirroring the enhanced levels of MHC class I and II observed in immunohistological analyses of IBM [22]. Complementing these findings, elevated levels of CD74—a protein critical for the formation and transport of MHC class II peptide complexes and subsequent T cell activation—were also observed (Fig. 2b).

Surprisingly, we also observed the upregulation of histone variants, with HIST2H2AA3 (also known as H2AC18) emerging as the most significantly increased protein in IBM, alongside a concurrent rise in histone H1FX levels. Although the functional roles of these histone modifications remain largely unexplored, their marked elevation in IBM suggests the possibility of epigenetic alterations that could contribute to disease pathogenesis. The most downregulated proteins in IBM included HSPB7—a heat shock protein involved in the cellular stress response—and PPP1R27, a regulatory subunit of protein phosphatase 1.

To further place these proteomic alterations within a biological context, we performed Gene Set Enrichment Analysis (GSEA) on the DEPs comparing IBM to non-IBM samples. Focusing first on the Gene Ontology (GO) biological pathways (BP) database, we found that pathways associated with antigen processing and the presentation of peptide antigens were significantly enriched in IBM. Additionally, pathways related to T cell-mediated immunity and activation were prominently upregulated, underscoring the role of this cell type in IBM. Conversely, pathways associated with mitochondrial function, energy metabolism, and RNA processing were significantly downregulated. A subsequent analysis using the GO cellular component (CC) database provided further insights into the tissue-level alterations in IBM. Here, an enrichment of proteins linked to the extracellular matrix and MHC complexes was evident, alongside a strong depletion of mitochondrial proteins.

Collectively, these findings reinforce the characterization of IBM as a disease with a strong T cell signature, marked by pronounced mitochondrial dysfunction and tissue remodeling.

### Analysis of the sPLS-DA candidate proteins

**Fig. 3:**
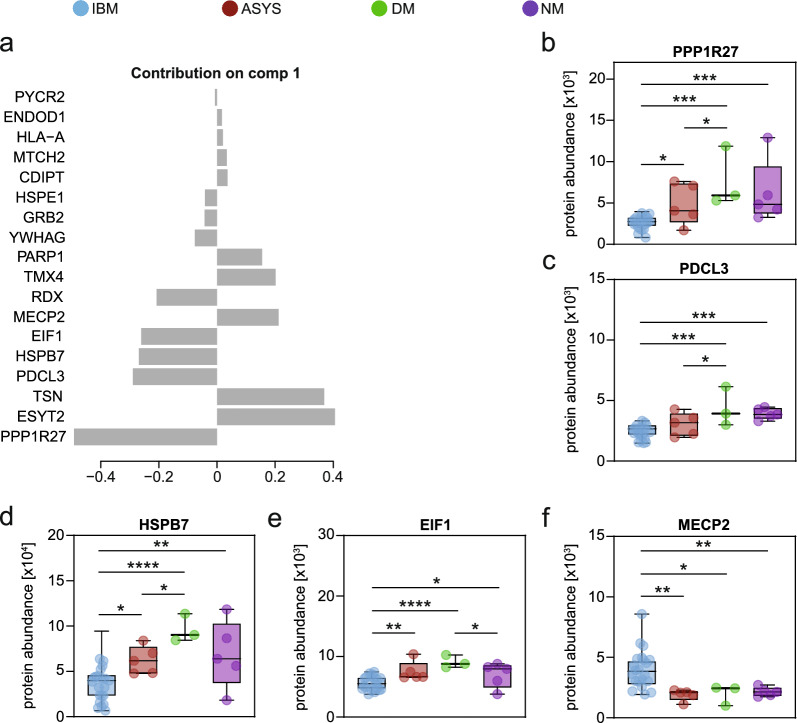
Analysis of individual proteins in the sPLS-DA model**. a** Contribution to component (comp) 1 of the sPLS-Da model. **b**–**f** Box-plots indicating the individual protein levels for each patient. Groups were compared by the ordinary one-way ANOVA test. *p* < 0.05 *, *p* < 0.01 **, *p* < 0.001 ***. *p* ≥ 0.05 not significant

Given the ability of sPLS-DA to differentiate IIM subtypes, we next scrutinized the proteins selected by this algorithm. Focusing on component 1, which primarily distinguishes IBM from other IIM forms, we evaluated each protein contributing to the classification (Fig. 3a). In IBM, several proteins involved in cellular regulation, cytoskeletal integrity, and muscle function showed prominent contributions. Notably, PPP1R27 emerged as the most substantial contributor, as also seen in the analysis above (Fig. 3b). Additionally, phosducin-like protein 3 (PDCL3), a chaperone critical for tubulin folding and cytoskeletal stability [23], was markedly downregulated in IBM, suggesting dysregulation of microtubule organization (Fig. 3c). Likewise, HSPB7, a protein integral to muscle maintenance and the cellular stress response [24, 25], was decreased in IBM (Fig. 3d). Eukaryotic translation initiation factor 1 (EIF1), which is essential for ribosomal translation initiation [26, 27], was reduced in IBM, potentially indicating compromised protein synthesis (Fig. 3e). Concurrently, methyl CpG binding protein 2 (MECP2) was strongly increased in IBM (Fig. 3f). As a gene regulatory element, MECP2 can enhance or repress the activity of other genes and its expression is required for skeletal tissue development [28]. By contrast, DM samples were characterized by reduced synaptotagmin-2 (ESYT2), a lipid transfer protein involved in membrane contact site formation [29], and translin (TSN) (Suppl. Figure 1), which plays a role in RNA transport and processing [30]. In summary, these findings underscore the heterogeneity of proteomic alterations across IIM subtypes. IBM is distinguished by the marked downregulation of proteins essential for maintaining cytoskeletal integrity, orchestrating stress responses, and regulating translation (including PPP1R27, PDCL3, EIF1, RDX, and HSPB7).

Taken together, the analysis of the cross-sectional cohort suggests that IBM has a distinct protein profile compared to other IIM subtypes. Among others, IBM is characterized by increased MHC class I and II levels, mitochondrial dysfunction, extracellular remodelling and T cell activity, thus aligning with the current knowledge on this disease. Moreover, we also describe a set of novel proteins specific to IBM, including markers of altered metabolism and epigenomic changes that warrant further research.

### Protein patterns in IBM and PM-Mito

**Fig. 4:**
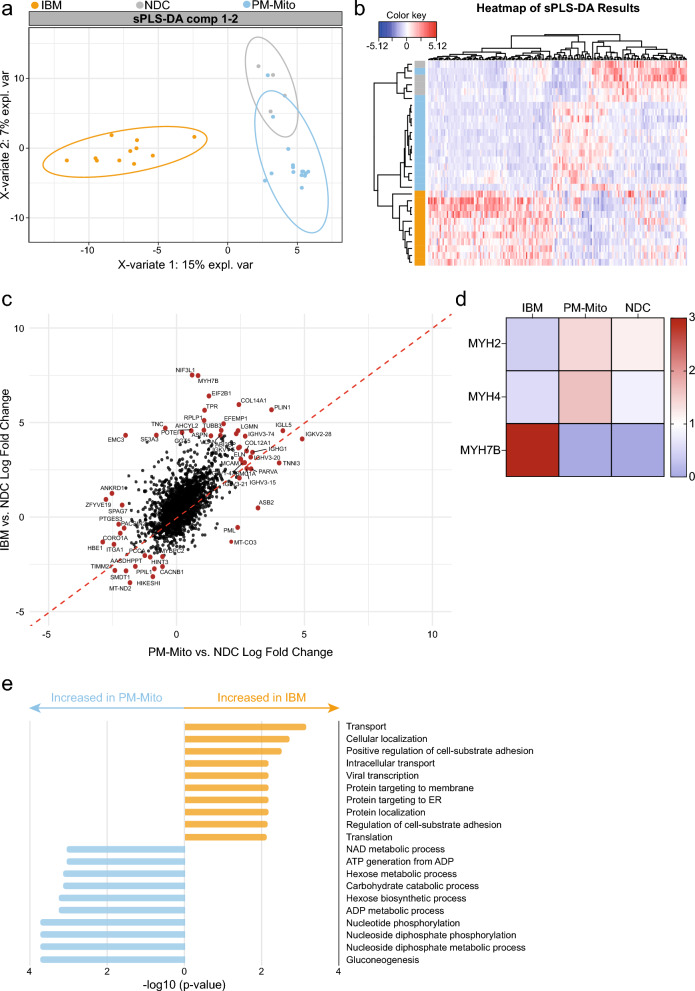
Protein profiles across the IBM spectrum. **a** Sample plots from the sPLS-DA model for the first and second component. **b** Clustered Image Map (CIM) of the data selected by sPLS-DA. Patients are depicted in rows, proteins in columns. The red and blue colours indicate the scaled protein levels. The dendrograms indicate the clustering of proteins and proteins, respectively. **c** Scatter plot indicating the fold-change of PM-Mito compared to NDC on the x-axis and the fold-change of IBM compared to NDC on the y-axis. Each dot is one protein. Proteins increased in both IBM and PM-Mito compared to NDC are on the top right. Proteins specifically increased in either IBM or PM-Mito are at the top left or bottom right of the plot, respectively. The top proteins are highlighted in red. **d** Heatmap indicating the scaled protein levels for each group. **e** Gene Set Enrichment Analysis (GSEA) for the GO Biological Processes dataset comparing IBM to PM-Mito. Proteins with a *p*-value ≤ 0.05 were chosen for the analysis

Next, we analyzed the IBM/PM-Mito cohort using sPLS-DA for dimensional reduction, as described above (Fig. 4a). In the final sPLS-DA model, the overall misclassification error rate was 0.016 ± 0.025 and the BER was 0.016 ± 0.025 (mean ± SD across repeats). This model yielded near perfect classification performances across groups, based on one-Vs-rest AUROC (mean ± SD): IBM 1.000 ± 0.000, NDC 0.994 ± 0.016, PM-Mito 0.998 ± 0.011. The final model retained a total of 37 proteins (Fig. 4b). This analysis revealed a separation between IBM and PM-Mito patients. NDCs exhibited overlap with PM-Mito, in accordance with histopathological findings, with some PM-Mito patients showing only very subtle tissue alterations. The overall protein patterns driving the sPLS-DA were distinctly different across groups. To further dissect the shared and unique proteomic alterations in IBM and PM-Mito, we compared protein levels in both conditions relative to NDCs (Fig. 4c). This approach allowed us to identify proteins that were shared in IBM and PM-Mito, as well as those uniquely altered in each condition. Plotting of IBM and PM-Mito compared to NDC, respectively, revealed a substantial number of proteins that were similarly increased or decreased in both disease groups (similarly increased in the top right quadrant, similarly decreased in the bottom left quadrant). It should be noted that the distinction in the sPLS-DA algorithm is driven by the selection of a set of highly differential proteins, while the overall changes to the proteome demonstrate a high degree of similarity between IBM and PM-Mito. Among the most upregulated proteins shared between IBM and PM-Mito were the protein peripilin (PLIN1), a key protein responsible for regulating lipid storage, and one of the immunoglobulin lambda-like polypeptides (IGLL5). COL14A1 was also substantially increased in IBM and PM-Mito. Interestingly, we [31] and others [32] previously reported that fibro-adipogenic progenitors (FAPs) assume a distinct phenotype in IBM characterized by the expression of *COL14A1* and *COL15A1*. Aligning with this observation, the shared increase in COL14A1 protein levels in IBM and PM-Mito suggests that an altered FAP phenotype might be present across both conditions. Conversely, the most downregulated proteins common to IBM and PM-Mito included the mitochondrial protein NADH dehydrogenase 2 (MT-ND2). MT-ND2 is the largest of the five complexes of the electron transport chain and dysfunctions in this gene have been implicated in mitochondrial diseases.

Besides their similarities, distinct proteomic markers also emerged for IBM and PM-Mito. Notably, MYH7B was among the most specifically increased proteins in IBM. MYH7B is a marker of type 1 myofibers, suggesting a transition from type 2 to type 1 myofibers in IBM, a phenomenon previously reported by us and others [31, 32]. Supporting this, type 2 myofiber markers MYH2 and MYH4 were more abundant in NDC and PM-Mito, whereas in IBM, their levels decreased, with a concomitant shift towards MYH7B as the dominant type 1 myofiber marker (Fig. 4d). In contrast, in PM-Mito, we observed a specific increase in proteins such as MT-CO3, a subunit of cytochrome c oxidase subunit III. Interestingly, alterations of cytochrome c oxidase subunit genes are associated with isolated myopathies [33]. Besides MT-CO3, the promyelocytic leukemia protein (PML) was also increased in PM-Mito compared to IBM or NDCs. PML is a major regulatory protein responsible, among others, for mediating apoptosis in a p53-dependent manner [34]. Additionally, the peroxiredoxin (PRDX) protein family exhibited alterations across the IBM disease spectrum (Suppl. Figure 2a). In particular, PRDX3 and PRDX5 were elevated in IBM compared to PM-Mito and NDC (Suppl. Figure 2b, c), suggesting that in IBM, these proteins might respond to oxidative stress. We also examined PPP1R27, given its differential levels across IBM and other IIM subtypes. While PPP1R27 was similarly downregulated in both IBM and PM-Mito relative to NDC, statistical significance was not reached due to high variability (Suppl. Figure 2d). Finally, we performed GSEA using the GO BP database to compare IBM and PM-Mito. This analysis highlighted distinct metabolic alterations in PM-Mito, including pathways related to gluconeogenesis, nucleotide phosphorylation, and carbohydrate catabolism. These findings indicate that, despite an overall intact skeletal muscle parenchyma, PM-Mito is characterized by substantial metabolic remodeling (Fig. 4e).

### Validation of proteomic findings in PM-Mito/IBM patients

**Fig. 5:**
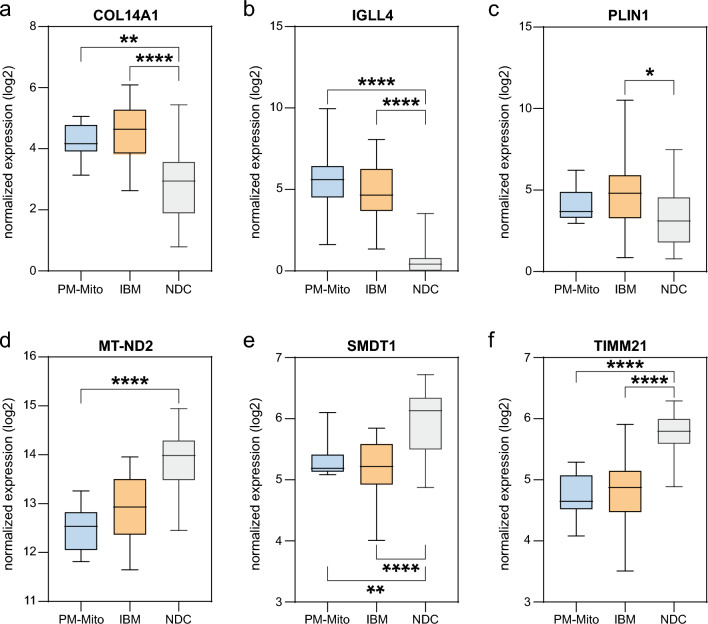
Validation of proteomic findings by bulk RNA sequencing. Box plots represent log-scaled normalized expression (log2(TMM+ 1)) levels of differentially expressed mRNA (A-F) in PM-Mito, IBM vs. non-diseased controls (NDC). Groups were compared by the ordinary one-way ANOVA test. *p* < 0.01 **, *p* < 0.001 ***, *p* < 0.0001 ****. *p* > 0.05 not significant

To corroborate our proteomic observations at the transcriptional level, we utilized a previously published dataset employing bulk RNA sequencing from muscle biopsies from IBM and PM-Mito patients [12]**.** We chose to focus on the most up- and downregulated proteins in the second cohort. Our analysis confirmed the dysregulation of transcripts corresponding to key proteins (Fig. 5a–f), including *COL14A1*, *IGLL4*, *PLIN1*, *MT-ND2*, *SMDT1*, and *TIMM21*. These alterations in mRNA levels mirror the change of protein abundances identified in our proteomic dataset, providing cross-platform validation of the molecular alterations in these myopathies. Notably, these results reinforce our proteomic findings and underscore the utility of integrating transcriptomic data via bulk RNA-seq to confirm disease-related protein dysregulation.

## Discussion

Our study provides a comprehensive proteomic characterization of IBM and its relation to other IIM subtypes, particularly PM-Mito. It reveals distinct molecular signatures that align with known pathological features while highlighting novel insights into disease mechanisms.

This analysis underlines that IBM is a distinct entity within the IIM spectrum, characterized by a combination of immune activation, mitochondrial dysfunction, and extracellular matrix remodeling. The pronounced upregulation of MHC class I and II molecules, including HLA-A and HLA-DRB1, and increased CD74 align with previous histopathological observations of IBM muscle fibers displaying MHC class I and II positivity and CD8^+^ T cell infiltration.

Our study builds on previous mass spectrometry-based proteomic studies in IIM. These have so far been limited to small patient cohorts (≤ 20 patients) and specific subtypes [35–37]. Early studies using 2D-PAGE and mass spectrometry in IBM [8] identified upregulated amyloid-related proteins, chaperones, and metabolic enzymes, as well as downregulation of muscle contraction proteins. More recent analyses using LC–MS/MS revealed increases of immune-related proteins such as CD74, CD163, and STAT1, implicating macrophage activation and interferon signaling as pathophysiological drivers [38]. Our data align with these findings, as we also observed a strong increase in CD74. Further, subcellular proteomics highlighted extensive alterations across nuclear, mitochondrial, and ER and Golgi compartments in IIM [39], while SILAC-based quantitative proteomics confirmed dysregulation of ~ 500 proteins, particularly in RNA metabolism and translation, with changes preceding histopathological signs [40]. It should be noted, however, that comparison of mass-spectrometry-based proteomics is confined to the dynamic range of the method employed by the respective study, and that the absence of protein alterations can also be attributed to limitations of detection.

The classification of PM-Mito remains debated; yet converging clinical, histopathological, and molecular evidence increasingly supports its inclusion within the IBM spectrum rather than its definition as a distinct entity. While the absence of rimmed vacuoles has often been used to differentiate PM-Mito from IBM, this feature is not pathognomonic, and may depend on biopsy site and sampling, as vacuoles can be patchy and may be absent in samples in early stages. Importantly, PM-Mito diagnosis does not rely solely on this feature but also considers other IBM-associated changes, including mitochondrial abnormalities, p62-positive aggregates, and distinct immune signatures [6, 12]. Consistent with longitudinal observations, 50 to 90% of PM-Mito cases eventually evolve into clinically or histopathologically defined IBM, supporting a shared disease continuum [4, 6]. Our proteomic and transcriptomic data further substantiate this concept, demonstrating an overlap between IBM and PM-Mito across mitochondrial, immune, and extracellular matrix pathways, indicative of a common pathogenic trajectory in which mitochondrial remodelling and immune activation precede structural muscle changes. Collectively, these findings argue that PM-Mito represents an early or incomplete manifestation of IBM, while recognizing that a minority of cases may remain clinically stable or partially responsive to immunotherapy.

Interestingly, both IBM and PM-Mito exhibited hallmarks of mitochondrial dysfunction at the protein level, yet IBM showed a pronounced increase in oxidative stress-related proteins PRDX3 and PRDX5. These proteins are involved in mitochondrial redox homeostasis, and their upregulation suggests a compensatory response to chronic oxidative stress within muscle cells [41]. Given the well-established link between mitochondrial impairment and IBM pathology, these findings further support mitochondrial dysfunction as a central contributor to disease progression.

Despite this considerable overlap, distinct proteomic markers differentiate IBM from PM-Mito. The upregulation of MYH7B in IBM and a concurrent decline in MYH2 and MYH4 support previous findings of a shift from type 2 to type 1 myofibers in IBM [31, 32]. In contrast, PM-Mito retains a balanced myofiber composition similar to NDC, aligning with a precursor state. This also aligns with the mild abnormalities that are evident at the histopathological level in PM-Mito [6]. We acknowledge the ongoing discussion on whether PM-Mito is a distinct disease entity or a precursor state to IBM. As between 50 and 90% of patients progress from PM-Mito to IBM, we suspect that at least a subset of patients belong to a shared disease continuum. Nonetheless, the rarity of the disease and the difficulty of detecting IBM during the early disease state add to the difficulty of finding a consensus for this discussion.

We also demonstrate the increase of histone variants HIST2H2AA3 and H1FX in IBM, a previously unreported feature of this disease. Given that histone modifications regulate gene expression, this raises the intriguing possibility that epigenetic dysregulation contributes to IBM pathogenesis. These findings warrant further investigation into whether chronic inflammatory signaling, oxidative stress, or metabolic stressors drive epigenetic alterations in IBM muscle. IBM also displayed cytoskeletal alterations, with downregulation of proteins crucial for structural integrity and cellular stability. The decrease in PPP1R27, PDCL3, HSPB7, and RDX suggests a loss of regulatory mechanisms that maintain muscle structure and function. PDCL3, a tubulin-folding chaperone, and RDX, an actin-binding protein, highlight the potential dysregulation of microtubule organization in IBM. Indeed, microtubule organization, as well as cytoskeleton stability, are linked to TDP-43. A loss of function of TDP-43 has been proposed as a cell-intrinsic feature of IBM in an elegant study employing a xenograft model of IBM [42]. Following this line of argument, the observed protein alterations might additionally reflect cell-intrinsic defects characterizing IBM.

While our study provides novel insights, a set of limitations must be acknowledged. The sample size for DM, ASyS, and IMNM was relatively small, limiting broader conclusions about these subtypes. Additionally, proteomics offers a snapshot of molecular changes; integrating transcriptomic and epigenomic analyses would provide a more comprehensive view of IBM pathophysiology. Future studies should also incorporate longitudinal patient data to track the PM-Mito-to-IBM transition better. Finally, we were limited to measuring both cohorts separately. However, to effectively place PM-Mito inside the IIM spectrum, a single measurement would be required, not to face batch effects that are difficult to overcome using proteomics, and to enable direct comparison of proteomic data between PM-Mito and other IIM.

Overall, our proteomic analysis reinforces IBM as a unique inflammatory myopathy with degenerative and metabolic features distinct from other IIMs. PM-Mito shares key molecular characteristics with IBM, supporting its classification within the IBM disease spectrum. We identify novel proteomic markers, including histone variants, cytoskeletal regulators, and oxidative stress proteins, that provide novel insights into the pathogenesis of IBM. These findings necessitate future research on mitochondrial health, oxidative stress, and early intervention in PM-Mito.

## Supplementary Information

Additional file1: **Suppl. Figure 1 a**–**d** Box-plots indicating the individual protein levels for each patient. Extension of Fig. 3. Groups were compared by the ordinary one-way ANOVA test. *p *< 0.05 *, *p *< 0.01 **, *p *< 0.001 ***. *p* ≥ 0.05 not significant.

Additional file2: **Suppl. Figure 2 a** Heatmap indicating the scaled protein levels for each group. **b–d** Box-plots indicating the individual protein levels for each patient. Groups were compared by the ordinary one-way ANOVA test. *p *< 0.01 **, *p* < 0.001 ***, *p *< 0.0001 ****. *p *> 0.05 not significant.

Additional file3 (PDF 506 KB)

## Data Availability

Mass spectrometry-based proteomic data have been deposited in the ProteomeXchange Consortium (https://repository.jpostdb.org/preview/8754332576821b299cc0db) via the jPost partner repository [43]. The accession numbers are PXD063841 for ProteomeXchange and JPST003801 for jPOST. Access key is: 8890.
