# Supplementary figures and images for: Proteomic profiles in inclusion body myositis and polymyositis with mitochondrial pathology
Source: Acta Neuropathol Commun. 2026 Feb 4;14:49. doi: 10.1186/s40478-026-02243-9 (PMC12930912; doi:10.1186/s40478-026-02243-9)

**a**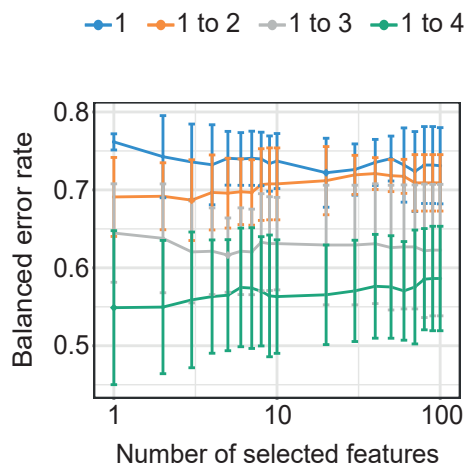**b**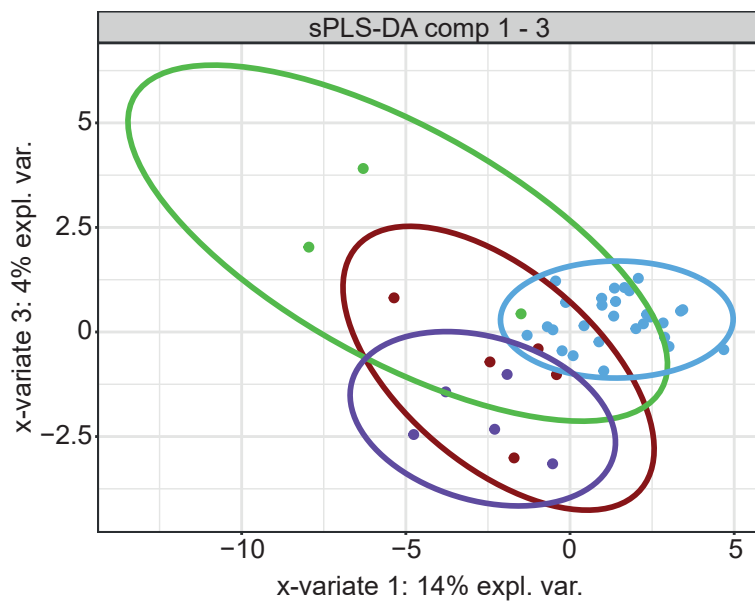**c**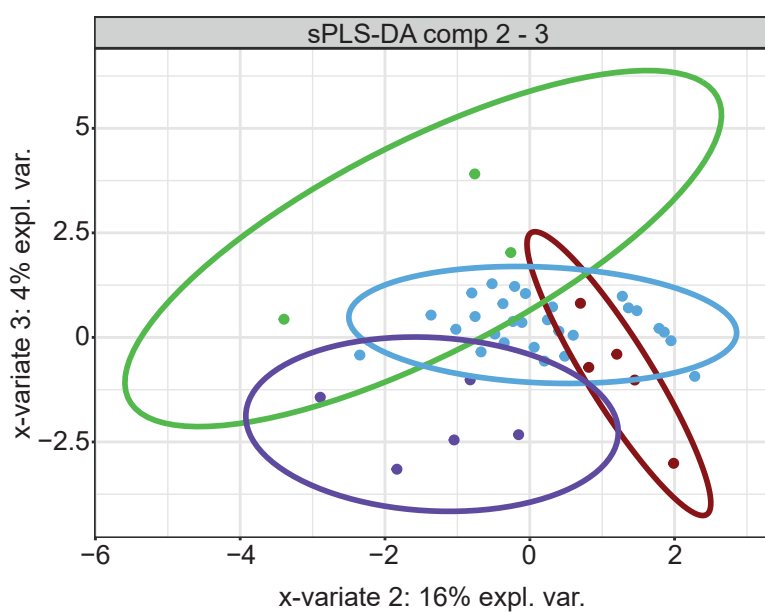

Supplement: Supplementary file 1 — Additional file1: Suppl. Figure 1 a–d Box-plots indicating the individual protein levels for each patient. Extension of Fig. 3. Groups were compared by the ordinary one-way ANOVA test. p < 0.05 *, p < 0.01 **, p < 0.001 ***. p ≥ 0.05 not significant. [file 40478_2026_2243_MOESM1_ESM.pdf]

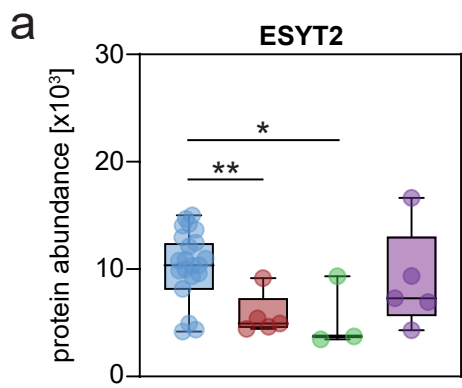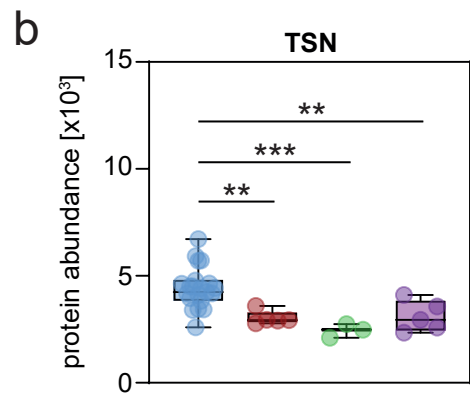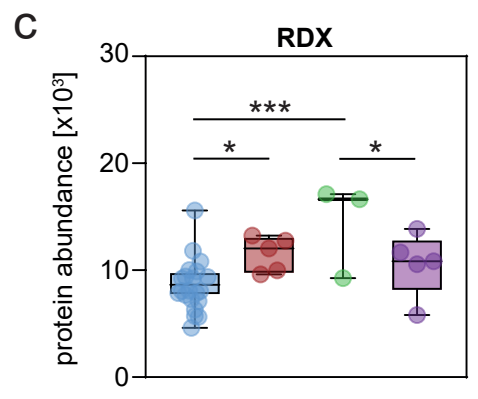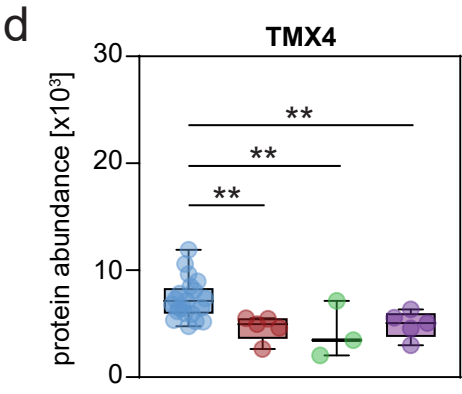

Supplement: Supplementary file 2 — Additional file2: Suppl. Figure 2 a Heatmap indicating the scaled protein levels for each group. b–d Box-plots indicating the individual protein levels for each patient. Groups were compared by the ordinary one-way ANOVA test. p < 0.01 **, p < 0.001 ***, p < 0.0001 ****. p > 0.05 not significant. [file 40478_2026_2243_MOESM2_ESM.pdf]

**a**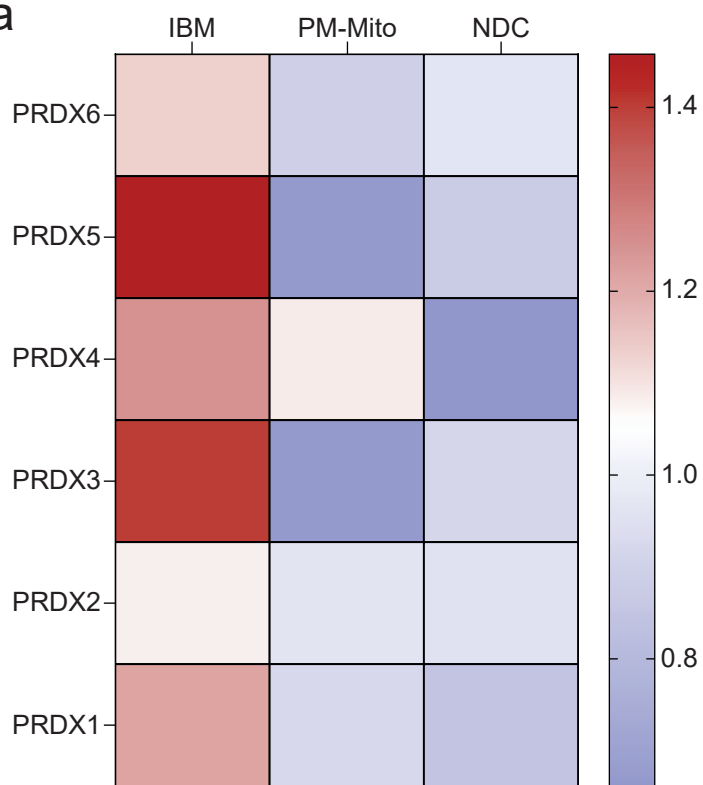**b**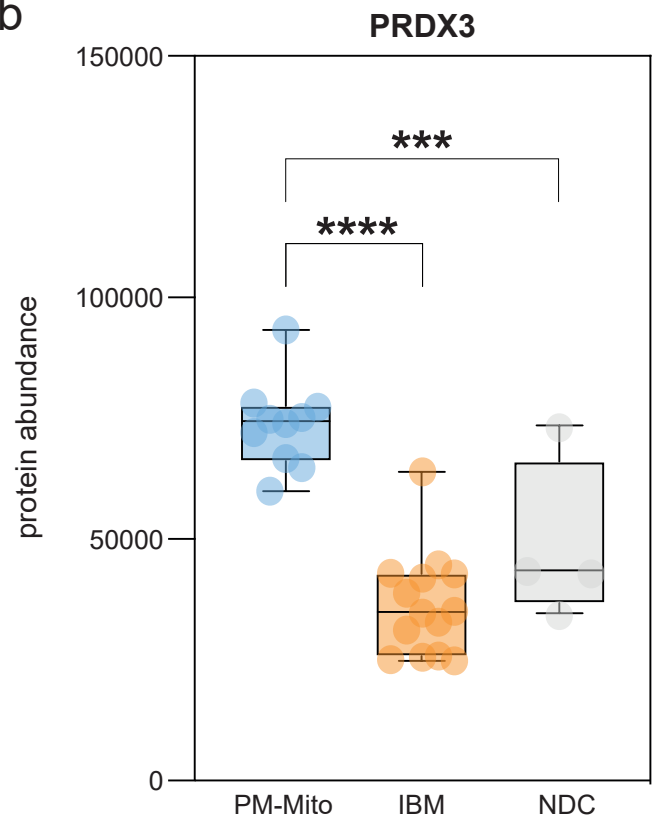**c**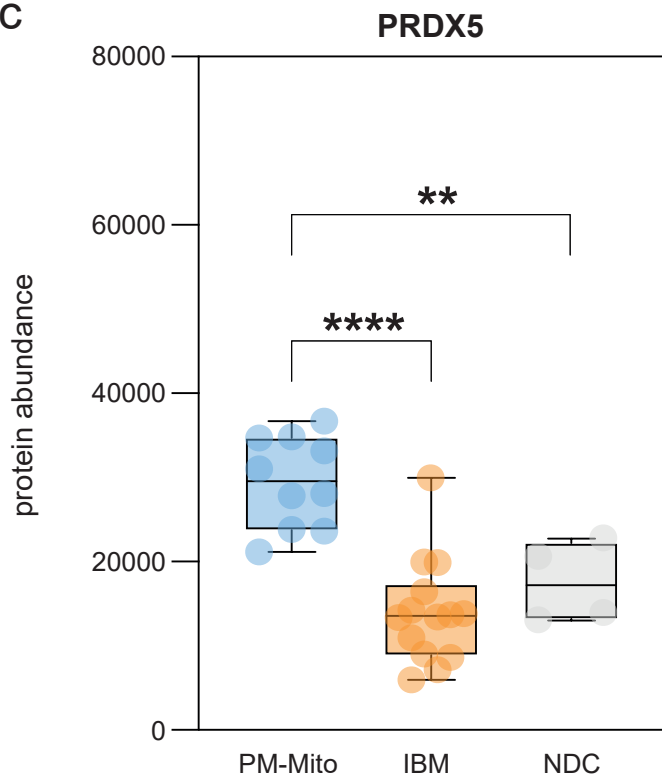**d**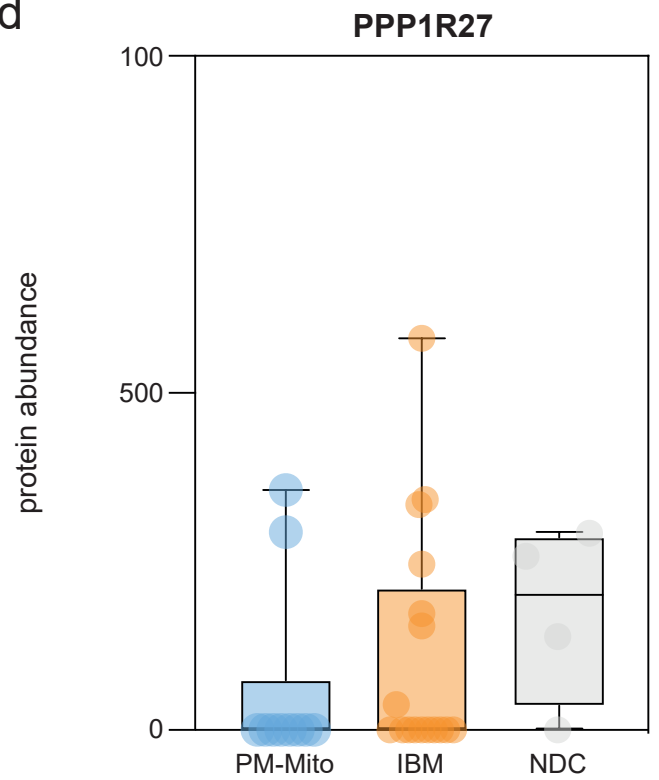

Supplement: Supplementary file 3 — Additional file3 (PDF 506 KB) [file 40478_2026_2243_MOESM3_ESM.pdf]
